# End-to-end contact enables long-distance electron transport between filaments in cable bacteria

**DOI:** 10.1093/ismejo/wraf194

**Published:** 2025-08-29

**Authors:** Rong Tang, Xiaoxue Zhang, Linyan Huang, Guoping Ren, Yin Ye, Yong Yuan, Shungui Zhou

**Affiliations:** Guangdong Key Laboratory of Environmental Catalysis and Health Risk Control, Guangzhou Key Laboratory Environmental Catalysis and Pollution Control, School of Environmental Science and Engineering, Institute of Environmental Health and Pollution Control, Guangdong University of Technology, Guangzhou, 510006, China; Guangdong Key Laboratory of Environmental Catalysis and Health Risk Control, Guangzhou Key Laboratory Environmental Catalysis and Pollution Control, School of Environmental Science and Engineering, Institute of Environmental Health and Pollution Control, Guangdong University of Technology, Guangzhou, 510006, China; Guangdong Key Laboratory of Environmental Catalysis and Health Risk Control, Guangzhou Key Laboratory Environmental Catalysis and Pollution Control, School of Environmental Science and Engineering, Institute of Environmental Health and Pollution Control, Guangdong University of Technology, Guangzhou, 510006, China; Fujian Provincial Key Laboratory of Soil Environmental Health and Regulation, College of Resources and Environment Fujian Agriculture and Forestry University, Fuzhou, 350002, China; Fujian Provincial Key Laboratory of Soil Environmental Health and Regulation, College of Resources and Environment Fujian Agriculture and Forestry University, Fuzhou, 350002, China; Guangdong Key Laboratory of Environmental Catalysis and Health Risk Control, Guangzhou Key Laboratory Environmental Catalysis and Pollution Control, School of Environmental Science and Engineering, Institute of Environmental Health and Pollution Control, Guangdong University of Technology, Guangzhou, 510006, China; Fujian Provincial Key Laboratory of Soil Environmental Health and Regulation, College of Resources and Environment Fujian Agriculture and Forestry University, Fuzhou, 350002, China

**Keywords:** cable bacteria, cooperative behavior, electron transport, Raman spectroscopy, electromicrobiology

## Abstract

Filamentous cable bacteria are capable of centimeter-scale long-distance electron transport and play crucial roles in the biogeochemistry of aquatic sediments. However, the mechanisms underlying long-distance electron transport remain incompletely understood. This study reports dynamic contacts between separate filaments of cable bacteria, enabling them to relay electrons between sulfidic and oxic zones. Video microscopy of motile filaments in a microchamber slide setup revealed that some filaments did not fully bridge the gap between the sulfidic and oxic zone, but made transient contact with each other. Contacts were always end-to-end and often occurred repeatedly, in which filaments always followed the same trajectory back and forth. The contact frequency gradually increased over the first 20 days, and then declined afterwards. About 5.5% of cable bacterium filaments were observed to engage in contact events during a 2-hour observation window on day 20. Confocal microscopy confirmed the presence of extracellular polymer substance trails between filaments, which appear to guide consecutive end-to-end contacts. *In situ* Raman spectroscopy showed that connections enabled redox continuity between reduced and oxidized filaments, thus suggesting inter-filament electron transfer during physical contact. This inter-filament electron transport represents a novel type of microbial cooperation, and appears to be a strategy for establishing optimal connections between spatially separated electron donors and acceptors in a dynamic sedimentary environment.

## 1. Introduction

Cable bacteria, belonging to the *Desulfobulbaceae* family, are multicellular microorganisms commonly found in both marine and freshwater sediments [1–5]. These organisms form dense populations at the sediment surface, and exhibit gliding motility that enables them to thrive within vertical redox gradients at the sediment-water interface [6–8]. One of the most remarkable features of cable bacteria is their capability for long-distance electron transfer (LDET), which allows them to connect zones of sulfide oxidation with regions of oxygen or nitrate reduction over centimeter-scale distances [9–12]. This ability differentiates them from other electroactive bacteria, which typically transfer electrons over much shorter distances, ranging from nanometers to a few hundred micrometers [13, 14]. The capacity for LDET is crucial for various biogeochemical processes in aquatic sediments. For instance, cable bacteria contribute to the acidification of anoxic sediments through electrogenic sulfur oxidation, thereby enhancing sulfur cycling and influencing trace metal dynamics [15–17]. Cable bacteria also facilitate the formation of iron oxides that sequester phosphates, thus providing a potential strategy for mitigating eutrophication in freshwater ecosystems [18–20]. In low-oxygen environments, cable bacteria can delay or prohibit the release of toxic hydrogen sulfide to the overlying water column, thus supporting aquatic life [21, 22]. Their unique electron transfer capabilities also present opportunities for improving conventional bioremediation by linking the degradation of pollutants in anoxic zones to oxygen at the sediment-water interface [23, 24]. Additionally, inoculating cable bacteria in rice cultivation is proposed to enhance sulfate levels and reduce methane emissions [25, 26].

Cable bacteria utilize elongated filaments to electrically connect the oxidation of free sulfide to the reduction of oxygen, processes that occur in separate cells positioned at opposite ends of these filaments [9, 27]. To ensure efficient electron transfer between these reactions, they employ a network of conductive protein fibers within the cell envelope, known as “cable” structures [28]. These fibers contain a sulfur-ligated nickel compound, thought to mediate electron transport, and share structural similarities with novel nickel complexes not typically observed in biological systems [29, 30]. Electrical measurements indicate that the fiber conductivity can exceed 100 S/cm, placing them among the most conductive biological materials known, surpassing a variety of organic semiconductors [31]. In conjunction with these conductive fibers, c-type cytochromes are believed to play a role in the LDET process [27]. A gradient in the oxidation state of cytochromes has been observed in intact bacteria, spanning from the sulfide oxidation zone to the oxygen reduction zone [32]. Despite these advancements in our understanding of cable bacteria, the mechanisms underlying LDET are still not fully elucidated, partly due to challenges with culturing and the physiological complexity of the LDET process.

In this study, our objective was to investigate any potential interactions between separate cable bacterium filaments. We tracked living cable bacterium filaments by microscopy in a microchamber setup, following an approach as detailed previously [6, 32]. One chamber was carefully filled with cable bacteria-rich sediment, whereas the other was filled with ambient air. To accurately identify cable bacteria, 16S RNA gene sequencing and fluorescence in situ hybridization (FISH) were utilized. A combination of optical microscopy and electron microscopy was utilized to confirm the length distribution patterns of cable bacteria in this particular microchamber setup and record their interaction behaviors among individual filaments. Furthermore, in situ Raman spectroscopy was applied to probe the oxidation states of c-type cytochromes within cable bacteria, thereby revealing whether electron transfer occurrs during contact between two filaments. This inter-filament interaction entails a novel, electrical type of cooperation within filamentous bacteria, which provides improved insight into the functioning of microbial and biogeochemical cycling in aquatic sediments.

## 2. Materials and methods

### 2.1. Sediment collection and incubation

Natural sediment was collected with a grab sampler from a freshwater river in Nancun, Guangzhou, China (23°0′18.14″N, 113°23′10.89″E) on September 3, 2022. The collected sediment was manually mixed, and then passed through a 0.5 mm mesh sieve to remove benthic animals and stones. The sediment was homogenized under a nitrogen atmosphere and transferred into glass tubes with an inner diameter 37 mm and a height 70 mm. Then, the sediment cores were transferred to a 50-liter tank filled with unfiltered tap water, where continuous aeration was provided by an air pump. The sediment cores were placed in a dark environment at 15 °C, and unfiltered tap water was added to the tanks weekly to compensate for evaporation loss. After approximately two weeks of cultivation, filamentous bacteria could be observed in the sediment using an optical microscope (Aote, BK6000).

### 2.2. Slide culture and microscope observation

**Figure 1:**
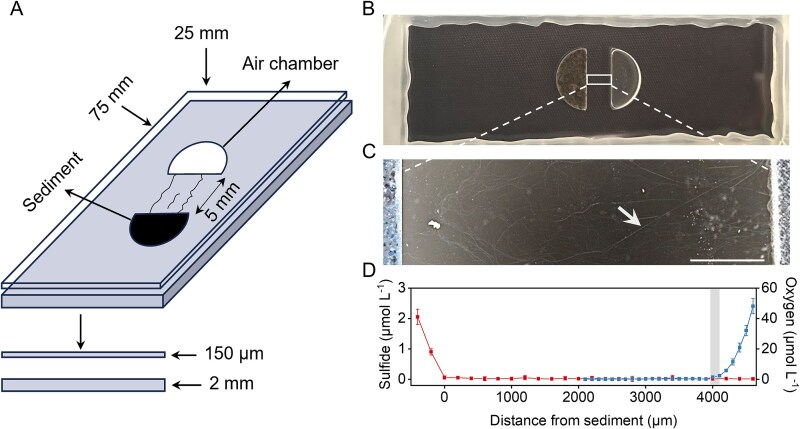
Enrichment of filamentous cable bacteria in the microchamber slide setup. (A) the setup contains two semicircular chambers grafted on a 2 mm thick glass slide, separated by a 5 mm space. One chamber is filled with sediment containing cable bacteria, the other chamber is filled with air. A 150 μm thick coverslip is then placed on the slide and sealed with hot-melt adhesive. (B) Image of the microchamber slide setup, the left semicircular chamber was filled with sediment (providing sulfide and cable bacteria) and the right semicircular chamber was filled with air (providing oxygen). (C) Dark-field microscopic image of the observation area in the microchamber slide setup, white arrows indicate cable bacterium filaments emerging from the sediment (left) and extending towards the air chamber (right). Scale bar, 1000 μm. (D) Concentration gradients of free sulfide (red) and oxygen (blue) across the slide setup as determined by microsensor profiling (n = 5). Gray shading indicates the microaerophilic veil.

To investigate the behavior of filamentous bacteria, a microchamber slide with two separated chambers was employed, constructed as described in a previous report [32]. In detail, two semicircular chambers were created in a glass slide (dimensions of 75.0 × 25.0 × 2.0 mm) using a glass laser precision cutting machine (Huanuo Laser Technology Co., Ltd.) separated by a distance of 5.0 mm. Each chamber had a diameter of 12.0 mm and a depth of 1.0 mm (Fig. 1A). After incubating the sediment for two weeks in the glass tubes, we collected the surface sediment and carefully rinsed it with ultrapure water to obtain a filamentous-enriched sediment aggregate. This aggregate was then transferred into the left semicircular chamber of the microscopy slide (sediment chamber), and the right chamber was filled with air (air chamber). Both chambers were covered with a single coverslip (75×25×0.15 mm). The space between the chambers was filled with ultrapure water flushed with nitrogen gas, and filter paper was used to remove excess sediment particles to ensure clear visibility during subsequent observations. Hot melt adhesive (DeliYC705, Deli Group Co., Ltd., China) was applied to seal the space between the bottom slide and the coverslip, thereby creating approximately 90 μm-thick water layer between the coverslip and the microchamber slide. Parallel slide setups (n=20) were prepared and incubated in the dark for approximately 48 hours, and then regularly imaged under a stereomicroscope with a 4× objective (Aote, SZ810) and using an optical microscope with 10×, 20×, and 40× objectives (Aote, BK6000).

### 2.3. Microsensor profiling

After the slide setup was incubated for approximately 24 hours in the dark, the concentrations of free hydrogen sulfide (H_2_S) and oxygen (O_2_) were measured using extralong microsensors with a tip diameter of 25 μm (Unisense, Denmark). The microsensors were mounted on a motorized micromanipulator and inserted into the water layer between the microchamber slide and coverslip, starting from the air chamber and moving towards the sediment chamber. Oxygen concentrations were measured at 100 μm intervals from the edge of the air chamber to 2 mm beyond the microaerophilic veil. For H_2_S, concentration profiles were recorded at 200 μm intervals from the edge of the air chamber to the interior of the sediment.

### 2.4. Fluorescence in situ hybridization

Fluorescence in situ Hybridization (FISH) was performed to identify the filamentous bacteria as cable bacteria. To this end, three microchamber slides were randomly selected after 48 hours of incubation. Subsequently, the hot melt adhesive was removed, and the microchamber was transferred to ultrapure water. The coverslip was then slowly opened, and filamentous bacteria aggregates were collected using a pipette. The filamentous bacteria were transferred to a confocal dish and fixed with 4% paraformaldehyde for approximately 3 hours. Then, 0.01 M PBS buffer solution was added, and the samples were washed three times to remove excess paraformaldehyde. The samples were dehydrated sequentially with 50%, 80%, and 96% ethanol solutions for 3 minutes each, followed by air-drying. A hybridization cocktail was prepared by mixing the Cy3-labeled *Desulfobulbaceae*-specific probe DSB706 (5′-ACCGGTATTCCTCCCGAT-3′), the FITC-labeled universal bacterial probe EUB338 I–III, and a standardized hybridization buffer (comprising 0.9 M NaCl, 20 mmol/L Tris-HCl, 40% formamide, and 0.01% SDS) at a volumetric ratio of 1:1:8. This formulation ensured a final probe concentration of 1 μmol/L for each component [33, 34]. The EUB338 I–III probe served as the positive control to allow the general detection of bacteria, and the DSB706 probe was specifically applied to identify the *Desulfobulbaceae* family members. The dehydrated sample was immersed in pre-warmed hybridization buffer and transferred to a dark, humid chamber for hybridization at 46 °C for 2 hours. After hybridization, the sample was covered with 4′,6′-diamidino-2-phenylindole (DAPI) and stained in the dark for 15 minutes, followed by washing off excess probes with ultrapure water. Finally, after the samples were air-dried in the confocal dish, they were imaged using a Zeiss LSM 800 confocal laser scanning microscope (CLSM, Carl Zeiss, Germany) with a 40× objective.

### 2.5. LIVE/DEAD and extracellular polymeric substances (EPS) staining

To verify the viability of the short filaments engaged in contacts, the slide setup was incubated for one week in the dark, after which the cover slip was carefully removed. The slide was then immersed for 15 minutes in LIVE/DEAD stain, a dual-fluorescence mixture of SYTO 9 and propidium iodide (LIVE/DEAD BacLight Bacterial Viability Kit, Thermo Fisher Scientific), following the manufacturer's instructions. The stained sample was imaged using a confocal laser scanning microscope (CLSM 800; Carl Zeiss, Germany) with a 20× objective. SYTO 9 (for live cells) was excited at 488 nm, and propidium iodide (for dead cells) was excited at 543 nm [35].

After the slide setup was incubated for 20 days in the dark, the cover slip was carefully removed, and the slide was fixed in 2.5% glutaraldehyde for 12 hours to preserve the integrity of cellular structures. Fluorescence staining of EPS was carried out using a combination of Nile Red, FITC, Calcofluor White, and Concanavalin A (Invitrogen, USA) to target lipids, proteins, β-D-glucopyranose polysaccharides, and α-D-glucopyranose polysaccharides, respectively [36]. Initially, 100 μL of 0.1 M sodium bicarbonate buffer (pH 9.0) was introduced into the space between the sediment and air chambers to ensure that amino groups remained in an unprotonated state. This was followed by the addition of 100 μL of FITC solution (1 g/L), which was then allowed to incubate at room temperature for 1 hour to stain the proteins. Next, 200 μL of Concanavalin A solution (250 mg/L) was applied and incubated for 30 minutes to label the β-D-glucopyranose polysaccharides. This step was succeeded by the addition of 200 μL of Calcofluor White solution (300 mg/L), with another 30-minute incubation period dedicated to highlighting the α-D-glucopyranose polysaccharides. The final staining step involved the introduction of 100 μL of Nile Red solution (10 mg/L), which was incubated for 10 minutes to visualize the lipids. After each staining step, the slide was thoroughly washed with phosphate-buffered saline (pH 7.2) to eliminate any excess dye. The stained samples were then examined under a confocal laser scanning microscope (CLSM LSM 800; Carl Zeiss, Germany) to observe the distribution and localization of biomolecules in EPS formation.

### 2.6. 16S rRNA gene sequencing

After 48 hours of incubation, six slide setups were randomly selected, and the coverslips were carefully removed. The filamentous bacteria from three slides were pooled into one sterile centrifuge tube using a pipette. DNA was extracted using the PowerSoil DNA Isolation Kit (MoBio Laboratories, Inc., CA), following the manufacturer's instructions. The full-length bacterial 16S rRNA gene was amplified via PCR using the universal primers 27F (5’-AGA GTT TGA TCC TGG CTC AG-3’) and 1492R (5’-TAC GGC TAC CTT GTT ACG ACT T-3’), and the PCR products were purified for PacBio sequencing [37]. Circular consensus sequencing (CCS) reads were generated using SMRT Link v8.0 with the parameters minPasses ≥ 5 and minPredictedAccuracy ≥ 0.9. Sample demultiplexing was performed using lima v1.7.0 based on barcode sequences, and primer trimming was conducted using cutadapt v2.7. Sequences outside the expected length range for 16S (1200-1650 bp) were discarded. Chimeric sequences were removed during the denoising process using the DADA2 plugin implemented in QIIME2 (version 2020.6) [38, 39]. Amplicon sequence variants (ASVs) were inferred using DADA2, and a relative abundance threshold of 0.005% of the total sequences was applied to filter out low-abundance ASVs. Taxonomic classification of ASVs was performed by aligning representative sequences against the SILVA 138 database [40]. A phylogenetic tree was constructed in MEGA 11 using the Neighbor-Joining method and the Kimura two-parameter model with 1000 bootstrap replicates, based on sequences obtained in this study and closely related reference sequences obtained from the National Center for Biotechnology Information (NCBI) database [41–43]. Finally, the phylogenetic tree of the 16S rRNA gene was visualized using the online software TvBOT (v2.6.1) [44].

### 2.7. Electron microscopy

After several days of dark incubation, end-to-end contacts between filaments were observed on the microchamber slide. Optical microscopy was used to locate two contacting filaments, and the location of their junction was carefully marked on the microscopic slide. The microchamber chamber slide was then dried in a 20 °C vacuum drying oven for 2 hours, after which the coverslip was removed. Subsequently, the slide was re-examined under a microscope to confirm the sustained contact between the filaments. Following this verification, the dried slide was mounted onto the scanning electron microscope (SEM) sample holder, followed by sputter-coating with a 5 nm gold layer (Vacuum, Japan). To investigate the structure of the terminal ends of the filaments, filaments were collected from sediment cores incubated for two weeks. The filaments were dehydrated through a graded ethanol series (30%, 50%, 70%, 90%, and 100%), and then critical point dried with carbon dioxide using a critical point dryer (Hitachi HCP-2, Japan). The dried filaments were secured onto an SEM sample holder and sputter-coated with a 5 nm gold layer. Imaging was performed using a field emission SEM (Hitachi SU8220, Japan) under high vacuum conditions (10^-8^ Pa) and low voltage (5 kV).

To prepare the sample for transmission electron microscope (TEM) measurements, filamentous bacteria incubated for one week in the microchamber setup were collected and fixed in 2.5% glutaraldehyde (2 mL, 4 °C, 12 h). After removing the fixative, the samples were rinsed three times with PBS buffer, each for 15 minutes. Next, they were fixed with 1% osmium tetroxide solution for 1 to 2 hours, followed by three rinses with PBS buffer [45]. The samples were sequentially dehydrated in 30%, 50%, 70%, and 90% ethanol, followed by two rounds of dehydration in 100% acetone, each lasting 20 minutes. Finally, the samples were transferred to a transmission electron microscope (TEM, Tecnai T12, ThermoFisher Scientific) operating at 80 kV for morphological observation and image acquisition. To examine the cross-sectional structure of cable bacteria, filaments that were incubated for one week were embedded in LR White resin with 0.9% benzoyl peroxide, then packed in gelatin capsules and placed in a 70 °C oven for 12 to 48 hours for polymerization. The resin blocks were removed for subsequent use. The embedded resin blocks of the samples were sliced into ultra-thin sections of 70-90 nm using an ultra-thin slicer (Leica, Austria), and the floating sections were then collected on copper TEM grids (Thermo Fisher Scientific). Next, the cross-sections were stained with uranyl acetate for 10 min, subsequently stained with lead citrate for 10 min and dry grids with filter paper. Finally, the grids were transferred to a transmission electron microscope (TEM, Tecnai T12, ThermoFisher Scientific) operating at 80 kV for image acquisition.

### 2.8. Filament tracking

During the 30-day incubation period in the microchamber slide setup, the OPTEC TP2000 camera with the accompanying software (ImageView) was used to record time-lapse videos and still images. Approximately two hours of video were recorded daily, to document contact events between two filaments. By adjusting the hue, saturation, and contrast, the image clarity was further enhanced. To compare the length and morphology of cable bacteria in anoxic and oxic zones, images were taken near the sediment chamber and the air chamber, respectively. Simultaneously, the positions of the contact point between filaments and their distances from the sediment were recorded. The contact frequency is calculated by recording the number of contact events in a microchamber slide setup within a 2-hour observation period and dividing this by the total number of cable filaments observed in the slide setups. These records may underestimate the number of cable bacteria involved in the contact event, as the contact processes might occur simultaneously at other locations whereas the video was not being recorded.

### 2.9. Confocal Raman microscopy

The Raman spectra of individual filaments were recorded in the microchamber setup using a LabRAM HR Evolution confocal Raman microscope (Horiba). The microscope was equipped with a 500 mW, 532 nm laser, a 300 grating with a spectral resolution of 2.8 cm^−1^, an Andor EM CCD detector suitable for 500-650 nm emission, a pinhole set to 250 μm, and a 50× magnification objective lens. The laser output was set to 25% of its maximum power (equivalent to 125 mW). Raman spectra were recorded along the length of cable bacteria filaments that emerged from the sediment chamber, including those that reached the air chamber and those that did not. Longitudinal scans were executed along the length of each filament at intervals of 1 mm. Given the substantial variation in filament lengths, each scan was designed to cover the entire filament length by incorporating 3-6 measurement points spaced at equal intervals. An exposure duration of 10-20 seconds was applied to each measurement point. During the measurement, filaments were positioned under high magnification (50× objective lens) with precise focusing at each measurement point. Filament positions were recorded before and after each scan to track positional changes. If filament movement was observed to compromise data accuracy, measurements were restarted from another appropriate position. For filament involved in contact, in situ Raman spectra were recorded before and after the contact between filaments emerging from the sediment chamber and those near the air chamber. The intensity of the 750 cm^−1^ band was used to assess the redox state of cytochromes [32, 46]. For comparative analysis, the band intensity at 750 cm^-1^ was normalized by subtracting the median of the baseline from 735 to 740 cm^−1^ and from 760 to 765 cm^−1^.

## 3. Results

### 3.1. Enrichment and identification of cable bacteria

**Figure 2:**
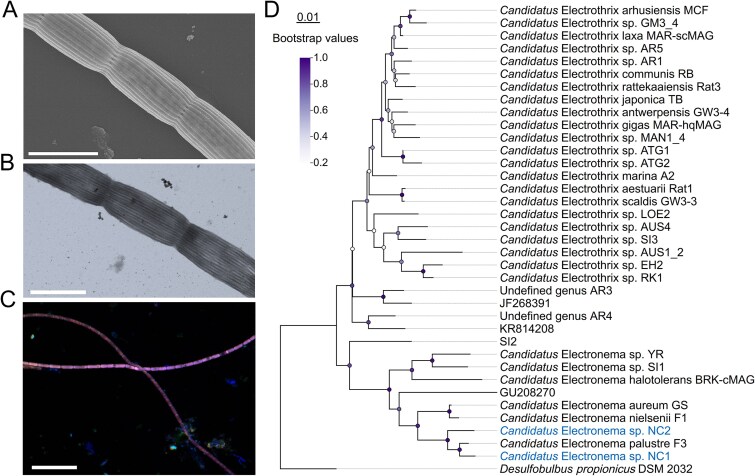
Morphological and phylogenetic identification of cable bacteria. (A) Scanning electron microscopy image of cable bacteria revealing the outer surface ridges. Scale Bar, 2 μm. (B) Transmission electron microscopy image showing the characteristic longitudinal ridges on the surface of cable bacteria. Scale bar, 2 μm. (C) FISH image with CY3-labeled DSB706 (red) and FITC-labeled EUB338 (green) probes, and DAPI (blue) as nuclear counterstaining. Scale bar, 20 μm. (D) Phylogenetic tree of cable bacteria based on 16S rRNA gene sequences obtained in this study (blue) and previously reported (black). Bootstrap values based on 1000 replications listed at branch points. *Desulfobulbus propionicus* DSM 2032 was used as the outgroup. Bar, 0.01 substitutions per nucleotide position. All accession numbers of the sequences used are listed in Supplementary Table S1.

After establishing the microchamber setup, stereomicroscope revealed that filaments had appeared from the sediment and established themselves within the gap between the sediment and the air chamber. Some filaments extended from the sediment into the oxygen-rich region, successfully bridging the gap between the sediment and air chambers (Fig. 1B and C). A microaerophilic veil was observed near the air chamber after two days of incubation (Fig. 1D and Supplementary Fig. S1). Microsensor profiling confirmed that both free sulfide and oxygen were undetectable within the region between the microaerophilic veil and the sediment edge, indicating the formation of a distinct suboxic zone over 4 mm wide (Fig. 1D). This redox zonation is consistent with previous profiling results in similar microchambers [32]. SEM imaging showed that filaments had parallel ridges on the outer surface (Fig. 2A), which is consistent with the unique morphology of cable bacteria [47, 48]. TEM imaging confirmed longitudinally extended, parallel ridges on the cable bacteria filaments (Fig. 2B). Using fluorescence in situ hybridization (FISH), the filamentous bacteria were identified as cable bacteria (Fig. 2C). A longitudinal cross-sectional image showed the connections between cells, whereas the transversal cross-sectional image at the cell midsection showed the ridges had a semicircular shape (Supplementary Fig. S3). In the transverse cross-section, the filament has 28 ridges with a diameter of 59 ± 7 nm, falling within the previously reported range (30-100 nm) of fiber dimensions [10, 48]. However, our observations by optical microscopy and electron microscopy indicated the presence of multiple filament morphotypes with varying diameters and ridge numbers (Fig. 2A, Supplementary Fig. S2 and Fig. S3). These morphotypes likely represent different strains/species or developmental stages.

Full-length 16S rRNA sequencing identified two complete 16S rRNA gene sequences belonging to the family *Desulfobulbaceae*. A phylogenetic tree was constructed based on these two full-length sequences combined with a relevant set of previously reported sequences that span the cable bacteria clade [5, 49]. The 16S rRNA gene sequence similarity between these two sequences obtained was 96.9%, below the bacterial species classification threshold (98.7%), indicating that they represented two distinct cable bacterium species [48]. Phylogenetic analysis revealed that both sequences belong to the genus *Candidatus* Electronema and clustered with *Candidatus* Electronema palustre F3 from freshwater sediments (Fig. 2D). Their 16S rRNA gene sequence similarities to F3 were 98.6% and 97.6%, respectively, both below the 98.7% threshold for species delineation, suggesting that they represent two potential novel species within the genus *Candidatus* Electronema. They are further referred to as *Candidatus* Electronema sp. NC1 and *Candidatus* Electronema sp. NC2.

### 3.2. Movement and positioning of cable bacterium filaments

Within 2 hours after establishing the microchamber slide incubation setup, cable bacteria started to emerge from the sediment chamber and accumulated near the sediment edge (Supplementary Fig. S4A and B), but no cable bacteria were observed near the air chamber. After 48 hours of incubation, more filamentous bacteria had emerged from the sediment in the microchamber slide. Most of these filaments had their terminal parts bent in half loops, which were then pushed forward by the rest of the filament towards the air chamber (Movie S1). After 15 days of incubation, an increased accumulation of cable bacteria within the suboxic zone, with numerous individuals remaining intertwined near the air chamber. At this time point, many cable bacterium filaments successfully spanned the zone between sediment and air chambers (Supplementary Fig. S4B). However, certain shorter filaments were unable to span this zone. After emerging from the sediment chamber, these filaments glided towards the oxic zone, where they gathered near the air chamber without returning to the sediment chamber (Supplementary Fig. S4B and Movie S2). The inability of certain filaments to span the suboxic zone can be attributed to their insufficient length and the intertwining that occurs near the air chamber, which may impede their positioning or movement. In parallel, numerous filaments, with lengths ranging from tens of micrometers to several hundred micrometers, were observed near the sediment chamber. These shorter filaments displayed parallel ridges on their cell surfaces (Supplementary Fig. S5A and B), had a similar cell size and morphology as the longer filaments, and were also identified as cable bacteria through FISH analysis (Supplementary Fig. S5C). Furthermore, LIVE/DEAD staining followed by CLSM visualization confirmed the viability of these short filaments (Supplementary Fig. S6). Short filaments were rarely observed near the air chamber (Supplementary Fig. S4B and Fig. S5D and E). The short filaments near the sediment chamber also displayed a relatively faster motility rate compared to those longer filaments located near the oxic zone. After approximately 30 days of incubation, some cable bacteria near the air chamber exhibited a darker color (Supplementary Fig. S4B), which may be due to diffusion of Fe^2+^ from the suboxic zone into the area near the air chamber, resulting in the precipitation of iron hydroxide oxide (FeOOH) onto the filaments [50].

### 3.3. Redox state of cytochromes in different types of filaments

**Figure 3:**
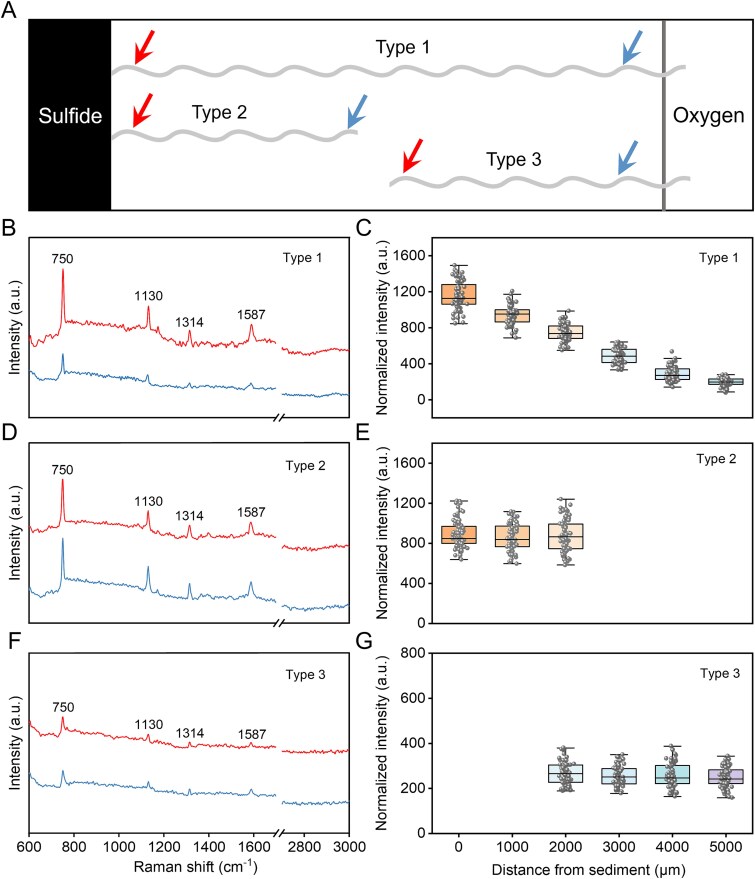
Raman spectroscopy enables assessment of the redox state of individual cable bacteria filaments. (A) Three types of filaments (light gray waves) with different connections toward sulfide and oxygen. Type 1 extends from the sediment (left) to the air chamber (right), whereas the other two filaments (type 2 and type 3) do not reach either the sediment or the air chamber. Raman spectra were recorded at positions indicated by the red (near the sediment) and blue arrows (near the air). (B) Raman spectra of type 1 filaments extending from the sediment to the oxygen. (C) Normalized intensity of the 750 cm^−1^ for type 1 (n=12 filaments). (D) Raman spectra of cable bacteria (type 2). (E) Normalized intensity of the 750 cm^−1^ band for type 2 (n=14 filaments). (F) Raman spectra of cable bacteria (type 3). (G) Normalized intensity of the 750 cm^-1^ band for type 3 (n=12 filaments). Six replicate Raman spectra were collected for each filament.

Individual filaments in the microchamber slide (incubated for 2-3 days) were analyzed using *in situ* Raman spectroscopy to assess the redox state of cytochromes. Three types of filaments were distinguished based on their “connection” state to the two chambers of the slide setup. One type of filament (Type 1) is capable of spanning both sediment and air chambers, whereas the other two have lost their connection to either the sediment or air chamber, respectively (Fig. 3A). All filaments displayed four prominent Raman bands characteristic of c-type cytochromes, located at 750 cm^−1^, 1130 cm^−1^, 1314 cm^−1^, and 1587 cm^−1^ [30]. Among these bands, the band at 750 cm^-1^, attributed to the pyrrole breathing mode of cytochromes, can be used as a key indicator for assessing the redox state of the cells [32]. For Type 1 filaments that extended from the sediment to the air chamber, the cells situated near the sediment end exhibited higher band intensity compared to those located near the air end (Fig. 3B). More detailed Raman spectra recordings revealed a gradual decrease in the intensity of the 750 cm^-1^ band from the cells located in the sediment region to those in the air region along the filament (Fig. 3C), indicating that the redox states of cytochromes transitioned from a reduced to an oxidized state, as observed previously [30, 32]. For those filaments that had no connection to the oxygen edge (Type 2; Fig. 3A), the cells at different ends showed no significant difference in Raman band intensity, with the intensity at 750 cm^-1^ remaining at a constant and relatively high value throughout the entire filament (Fig. 3D and E). This suggests that the cytochromes are in a reduced state, having received electrons resulting from sulfide oxidation. Similarly, for the filaments near the air chamber (Type 3; Fig. 3A), the terminal ends also showed no variation in Raman band intensity (Fig. 3F and G), but the band intensity was significantly lower than for the Type 2 filaments. This finding suggests that the cytochromes are in an oxidized state, having lost electrons by oxygen reduction.

### 3.4. Visualization of contact behavior

**Figure 4:**
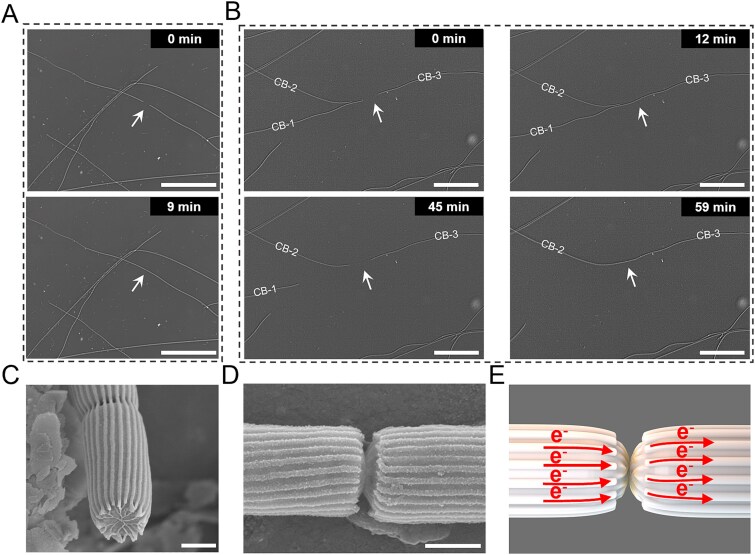
Observations of end-to-end contact between cable bacteria filaments. (A) Optical microscopic imaging showed two separate filaments establishing contact via an end-to-end connection. Scale bars, 50 μm. Two stills taken from movie S2. (B) Optical microscopic image illustrating how two separate filaments CB-1 and CB-2 progressively move toward filament CB-3 to initiate physical contact. Scale bars, 50 μm. Stills taken from movie S3. (C) SEM image showing the characteristic parallel ridges on the surface of bacteria, and the cartwheel-like structure at the interface between two cells. Scale bar, 500 nm. (D) High-resolution SEM image of two filaments that have been trapped during contact. Scale bar, 500 nm. (E) Diagram illustrating the physical interaction between two individual filaments, depicting their contact at the central node of the cartwheel structure. Red arrows denote the direction of electron transfer.

After the microchamber setup was assembled, videos were recorded daily for ~2 hours using an optical microscope (Aote, BK6000) to document the movement of cable bacteria within the setup. In these videos (Movie S2), the left side is near the sediment chamber, whereas the right side is near the air chamber. In one video, two individual cable bacterium filaments were observed to move towards each other and establish contact via an end-to-end connection at the 9th minute (Fig. 4A and Movie S2). The connected cable bacteria subsequently separated at the 23rd minute, thus providing a contact period of 14 minutes. In another video, the contact and subsequent separation were observed to involve multiple filaments. Three separate filaments were involved, designated as CB-1, CB-2, and CB-3 (Fig. 4B and Movie S3). During the first 12 minutes of the video recording, filament CB-1, situated at the sediment side, moved toward filament CB-3, which was located near the air chamber, and successfully made a connection. Subsequently, at the 20th minute, the filaments began to separate again, with the entire process of contact lasting approximately 8 minutes. After the separation was complete, filament CB-1 glided back toward the sediment side and disappeared from view by the 52nd minute. At the same time, filament CB-2 moved toward filament CB-3 and completed a contact before the video ended (Movie S3). Filament CB-3 hence engaged in a contact with both filament CB-1 and filament CB-2, thus indicating that the contact between cable bacterium filaments lacks specificity and is flexible in terms of contact partners. In one instance we observed that a short cable bacterium filament migrated toward a long cable bacterium filament, thereby establishing an initial contact (Supplementary Fig. S7 and Movie S4). Subsequently, separation occurs as the short filament returns to the left side of the field of view. Following this, the short filament moved once again toward the long filament, thus establishing a second contact. This observation indicates that the processes of contact and separation is a repeatable phenomenon. As observed above, the microchamber slide contained multiple filament morphotypes exhibiting variations in both diameter and ridge count. Yet, in all our observations, it appears that inter-filament contact always occurs within the same morphotype.

To further understand the contact behavior of cable bacteria, we quantified the frequency of contact as the number of contact events in a 2 hour period observed normalized by the number of filaments in the slide. On the 5th day of incubation, we observed the first contacts between separate filaments (Supplementary Fig. S8A). The frequency of contact gradually increased in the first 20 days, reached a maximum value of 5.5% on day 20 and then decreased (Supplementary Fig. S8B). The majority of inter-filament contacts occurred within 3 mm from the sediment chamber.

The structural features of the filaments involved in contact events were further investigated through SEM. Filaments were “trapped” during contact events, then fixed and coated with a gold layer for SEM analysis. The terminal end of the filaments displayed a prominent cartwheel structure that interconnects the individual fibers to a central node (Fig. 4C), a structure similar to those observed in previous studies [28, 51]. The SEM images indicated that the two filaments engaged in contact are directly connected end-to-end to form a junction through the cartwheels (Fig. 4D).

### 3.5. Electron transport between individual cable bacteria through physical contact

**Figure 5:**
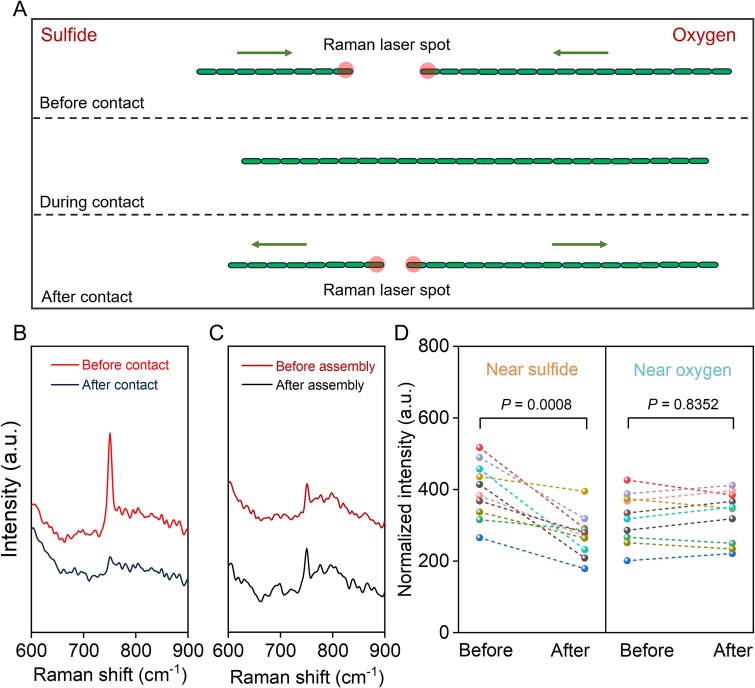
Changes in the redox state of cytochromes before and after contact. (A) Schematic of the Raman measurement positions. The red shaded circles indicate the Raman laser spots, and the green arrows represent the direction of filament movement. (B) Change in intensity of the 750 cm^-1^ band for filament near the sulfide front before and after contact. (C) Change in intensity of the 750 cm^-1^ band for filaments connected to the oxygen front before and after contact. (D) Normalized intensity change of the 750 cm^-1^ band for filaments before and after contact. The redox state of cytochromes in filaments located near the sediment experienced a significant change (n=10). The redox state of cytochromes in filaments located near the oxygen did not experience significant changes (n=10). *P* values were calculated using a two-sided *t*-test. A.u. = arbitrary units.

To investigate whether cable bacteria can transfer electrons between filaments via physical contact, we used Raman microscopy to analyze the redox state of cytochromes before and after the contact. A schematic diagram depicts the location and conditions for the Raman measurements (Fig. 5A). The terminal cells of the individual filaments were examined by measuring their Raman signal before and after the contact process. As done above, the band at 750 cm^-1^ was chosen as the representative indicator of the cytochrome redox state [32]. After contact, the intensity of the 750 cm^-1^ exhibited a significant decrease for the filaments that were located near the sulfide zone (Fig. 5B and D). The initially high band intensity suggests a reduced state of the cytochromes, resulting from the acquisition of electrons through the oxidation of sulfide present in the sediment. The decrease in band intensity upon contact suggests that electrons are subsequently transferred to the filament located near to the oxygen-rich side. However, the band intensity of the partner filament near the oxygen-rich side exhibited no significant change before and after contact (Fig. 5C and D). This may be attributed to the continuous exposure to oxygen, which causes the cytochromes in these filaments to be rapidly oxidized, thereby making it difficult to capture the transient reduced state using Raman spectroscopy.

## 4. Discussion

The microscopic chamber setup has previously been demonstrated to be highly effective to document the movement and positioning of cable bacterium filaments in relation to the redox gradient between sulfide and oxygen [32]. Consistent with previous observations [32], a suboxic zone formed within the slide setup between the sediment and air chambers, which is devoid of any measurable H_2_S and O_2_ (Fig. 1D). On the slide setup, a microaerophilic veil formed approximately 200 to 300 μm away from the air chamber after 48 hours (Supplementary Fig. S1 and Fig. S4B), indicating the presence of an anoxic–oxic interface [32, 46, 52]. After approximately one week of incubation in the dark, the microaerophilic veil disappeared, likely due to a shift of the oxic-anoxic interface toward the air chamber. At this time point, the cable bacteria began to extend into the air chamber to be able to access oxygen, and many individuals remained intertwined near the air chamber, consistent with previous observations [6, 32].

**Figure 6:**
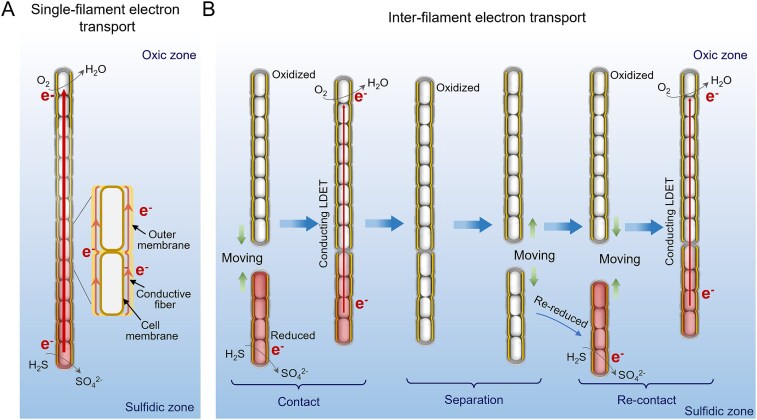
The contact and separation behaviors and the associated electron transport strategy in cable bacteria. (A) Single filament electron transport. Filaments can bridge the oxic and anoxic zones, directly facilitating LDET. (B) Inter-filament electron transport. Cable bacteria that lose connection with either the sulfide or the oxygen fronts perform electron transport through contact and separation of two individual filaments, thereby achieving LDET. The red color denotes the reduced state, whereas the white color represents the oxidized state of the cells.

Microscopic observations showed end-to-end physical contact between two filaments (Fig. 4), which primarily occurred within the suboxic zone at a distance of 1-3 mm from the sediment chamber (Supplementary Fig. S8). It implies that the specific conditions within the suboxic zone play a crucial role in facilitating the inter-filament contacts. Some individual cable bacterium filaments are capable of physically spanning the entire suboxic zone, thus enabling to conduct electrons all the way from the sediment chamber (sulfidic zone) towards the air chamber (oxic zone) [1, 28]. However, our results show that some long filaments are accumulating near the oxic zone, but have lost connection to the sulfidic zone (Supplementary Fig. S4). For these filaments that have lost the connection between distant electron donors and acceptors, we propose a novel electron transport strategy based on the end-to-end contact behavior between two individual filaments (Fig. 6). This mechanism involves shorter cable bacterium filaments relaying electrons between the sulfidic zone and the longer filaments that are in contact with the oxic zone.

Our Raman data reveal that the cytochromes of the shorter filaments have a reduced signature, whereas those of the longer filaments have an oxidized signature. This suggests that the two filament types establish contact via an end-to-end connection, enabling electron transfer from the reduced filament to the oxidized filament. Following the completion of electron transport, the connected filaments separate again by moving in opposite directions. This contact process can be repeated with the same partner or subsequent connections may also involve different partners (Movie S3). The duration of the inter-filament contacts was observed to last between 8 and 14 minutes. A simple model calculation shows that the length of this contact period matches the time needed for electron discharge. In Movie S4, the full length of the contacting filament on the sulfide side is ~60 μm. A discharge duration associated with this inter-filament contact event was estimated to be approximately 10.5 seconds (Supplementary Text S1). The actual contact duration was approximately 16 seconds, which is in close agreement with the estimated value. This provides supporting evidence for the physical plausibility of inter-filament electron transfer. The contact duration of the filaments in Movie S4 was much shorter than in Movie S3, as the reduced filament involved in end-to-end contact was significantly shorter than that in Movie S3.

The formation of a unique cartwheel at the cell-cell junctions of cable bacteria has been documented in earlier studies [28, 51]. This cartwheel structure is thought to enhance the redundancy and fail-safe nature of the internal electrical network within cable bacteria. The cartwheel consists of a set of spokes, which all start from a periplasmic fiber and radiate inwards, converging towards a central node [51]. In contrast to typical cell-cell connections in cable bacteria, which feature a continuous outer membrane with parallel longitudinal ridges [1, 48], the transient junction formed through direct filament contact exhibits a discontinuous outer membrane. Based on these observations, we propose a model for the end-to-end contact of two cable bacterium filaments (Fig. 4E). Filaments move towards each other and come into contact at the central node of the cartwheel. In this view, the central node functions as a crucial channel for this direct electron transfer. In contrast to indirect electron transfer mediated by redox mediators, this contact-dependent process enables direct electron transfer pathway between two individual filaments [46].

One conspicuous observation is the repeated contact between two filaments, where the shorter filament always follows the same trajectory (Movie S4). The excretion of EPS may play a crucial role in driving this repetitive contact between two filaments. The gliding movement of cable bacteria has been demonstrated to be closely associated with EPS excretion, and an EPS trail is left on a microchamber slide as cable bacteria move [50]. This association between EPS excretion and motility is not exclusive to cable bacteria, but has also been observed in other filamentous bacteria, such as cyanobacteria [53]. We identified EPS trails between individual cable bacteria on the slide via optical microscopy (Supplementary Fig. S9A and B). This observation was confirmed through CLSM with multiplex EPS stains targeting lipids, proteins and polysaccharides. This analysis clearly showed a distinct EPS trail between two filaments (Supplementary Fig. S9C-H). In light of these findings, EPS may be a key factor guiding the precise contact between two filaments.

Our study demonstrates that cable bacterium filaments can collaborate through spontaneous and precise end-to-end contact behavior, effectively achieving inter-filament LDET. This cooperative mechanism underscores the significance of filament interactions in optimizing electron transport within microbial communities and highlights the complex dynamics of these microorganisms in their natural habitat. When this collaborative behavior occurs in natural sediments, the electron transport distance of cable bacteria may be greater than expected based on filament length. Motility is crucial for cable bacteria as they navigate the shifting sulfide front, allowing them to maintain contact with oxygen despite the widening gap caused by their metabolism [6, 46]. When disconnected from either front, their motility enables reconnection. Cable bacteria typically stop gliding in oxygen-rich environments and resume movement when oxygen levels drop, a behavior consistent with filamentous sulfide-oxidizing bacteria that utilize motility and oxygen chemotaxis for optimal positioning [6, 54, 55]. The energy expenditure associated with motility is anticipated to be considerable for cable bacteria, given their need to traverse substantial distances to access sulfide or oxygen in dynamic environments. Although the precise energy expenditure for motility in cable bacteria remains unknown, insights from studies on other bacterial species can provide an estimation. For instance, type IV pili-mediated twitching motility in *Pseudomonas aeruginosa* can consume up to 10% of the cellular total energy budget [56]. Similarly, in *Escherichia coli*, motility has been shown to utilize up to several percent of total energy budget [57]. Considering their larger size and structural complexity, filamentous bacteria, such as cable bacteria, may allocate an even greater proportion of their energy budget to motility compared to non-filamentous counterparts. Therefore, the considerable energy expenditure associated with gliding may restrict the ability of cable bacteria to thrive in various environments. In contrast, the inter-filament electron transport model proposed here, which involves contact interaction between reduced and oxidized cable bacteria, provides a way for cable bacteria to address such distance-related challenges. Rather than migrating back to the anoxic sulfide zone to acquire electrons, cable bacteria in the oxic zone can directly obtain electrons from other reduced filaments. This contact-mediated electron transfer enables filaments to collaborate through transient contact and spontaneous separation, thereby sustaining metabolism and survival without returning to electron-donor zones. Such cooperation not only enhances the efficiency of electron transport but also reduces the reliance on energy-intensive motility, allowing cable bacteria to better conserve energy, thereby supporting their survival and maintaining ecological functions.

## 5. Conclusion

This study has uncovered a novel inter-filament interaction in microbiology, where cable bacteria can form end-to-end connections through highly precise physical contact. Microscopic observations revealed that a substantial fraction of the cable bacteria population frequently engages in such end-to-end interactions on a microchamber slide setup. Our observations also captured a distinct gliding motion of filaments along EPS trails during the contact, suggesting that EPS may play a pivotal role in guiding the precise alignment and contact between individual filaments. Significant changes in the redox state of c-type cytochromes were recorded during the contact event using Raman microscopy, providing compelling evidence for an active inter-filament electron transfer process. This unique inter-filament interaction is believed to enable the extension of long-distance electron transfer beyond the confines of individual filaments. These findings provide valuable insight into the collaborative behaviors of filamentous bacteria and advance our understanding of microbial electron transport mechanisms.

## Supplementary Material

Supplementary_material_wraf194

Movie_S1_wraf194

Movie_S2_wraf194

Movie_S3_wraf194

Movie_S4_wraf194

## Data Availability

All relevant data from this study are available in the article, and/or supplementary material. The sequences related to cable bacteria obtained in this study have been uploaded to the NCBI database, with accession numbers PQ351298-PQ351299. The sequencing data have been uploaded to the NCBI Sequence Read Archive (SRA) under the BioProject accession number PRJNA1270696.
