# Supplementary material for: End-to-end contact enables long-distance electron transport between filaments in cable bacteria
Source: ISME J. 2025 Aug 29;19(1):wraf194. doi: 10.1093/ismejo/wraf194 (PMC12449579; doi:10.1093/ismejo/wraf194)
Supplement: Supplementary_material_wraf194 [file supplementary_material_wraf194.docx]

**End-to-end contact enables long-distance electron transport between filaments in cable bacteria**

Rong Tang^1^, Xiaoxue Zhang^1^, Linyan Huang^1^, Guoping Ren^2^, Yin Ye^2^, Yong Yuan^1,^*, and Shungui Zhou^2,*^

^1^Guangdong Key Laboratory of Environmental Catalysis and Health Risk Control, Guangzhou Key Laboratory Environmental Catalysis and Pollution Control, School of Environmental Science and Engineering, Institute of Environmental Health and Pollution Control, Guangdong University of Technology, Guangzhou 510006, China

^2^Fujian Provincial Key Laboratory of Soil Environmental Health and Regulation, College of Resources and Environment, Fujian Agriculture and Forestry University; Fuzhou, 350002, China.

* Corresponding Author: Yong Yuan and Shungui Zhou

Email addresses: [yyuan2017@gdut.edu.cn](mailto:yyuan2017@gdut.edu.cn) (Yuan Y.); [sgzhou@fafu.edu.cn](mailto:sgzhou@fafu.edu.cn) (Zhou S.)

**Text S1. An estimation of the duration of discharge during inter-filament contact.**

The inter-filament contact event observed in Movie S4 was analyzed based on the following assumptions and quantitative calculations. The filament involved in the contact near the sediment side is approximately 60 µm long and contains 28 fibers each with a diameter of about 50 nm. Assuming a density of one Ni/S cofactor per nanometer along each fiber (with each cofactor storing one electron) [1], the charge storage capacity of a 60 µm-long filament can be estimated as follows:

Q = 60,000 nm × 28 × π × (50)^2^ / 4 ≈ 3.3×10^9^ electrons

Assuming a similar capacitance contribution from periplasmic components (e.g., cytochromes), the total charge storage capacity may double to ~6.6×10⁹ electrons. Given a continuous discharge current of 100 pA (i.e., ~ 6.3×10⁸ electrons/sec) [2, 3], this would imply a discharge duration of:

τ = Q / I ≈ 6.6×10⁹ / 6.3×10⁸ ≈ 10.5 seconds

**Table S1.** **The accession numbers of the 16S rRNA genes** **sequences used in the phylogenetic tree of Figure 2.**

| **Species** | **Accession number** |
| --- | --- |
| *Ca*. Electrothrix gigas MAR-hqMAG | GCA_942491745 |
| *Ca.* Electrothrix japonica TB | GCA_026122855 |
| *Ca*. Electrothrix sp. MAN1_4 | GCA_022766125 |
| *Ca*. Electrothrix sp. ATG1 | GCA_022765905 |
| *Ca*. Electrothrix sp. ATG2 | GCA_022765965 |
| *Ca*. Electrothrix arhusiensis MCF | GCA_004028505 |
| *Ca*. Electrothrix sp. GM3_4 | GCA_022765805 |
| *Ca*. Electrothrix communis RB | GCA_030644725 |
| *Ca*. Electrothrix sp. AR1 | GCA_022765785 |
| *Ca*. Electrothrix sp. AR5 | GCA_022765745 |
| *Ca*. Electrothrix laxa MAR-scMAG | GCA_942492895 |
| *Ca*. Electrothrix marina A2 | GCA_004028515 |
| *Ca*. Electrothrix rattekaaiensis Rat3 | GCA_032595675 |
| *Ca*. Electrothrix sp. LOE2 | GCA_022766145 |
| *Ca*. Electrothrix sp. AUS1_2 | GCA_022765865 |
| *Ca*. Electrothrix sp. AUS4 | GCA_022765925 |
| *Ca*. Electrothrix aestuarii Rat1 | GCA_032595685 |
| *Ca*. Electrothrix sp. EH2 | GCA_022765845 |
| Undefined genus AR4 | GCA_022765725 |
| Undefined genus AR3 | GCA_022765765 |
| *Ca*. Electrothrix sp. SI3 | PQ214941 |
| *Ca*. Electrothrix sp. RK1 | PQ214938 |
| *Ca*. Electrothrix antwerpensis GW3-4 | CP147990 |
| *Ca*. Electrothrix scaldis GW3-3 | CP138355 |
| *Ca*. Electronema halotolerans BRK-cMAG | GCA_942493095 |
| *Ca*. Electronema aureum GS | GCA_004284765 |
| *Ca*. Electronema nielsenii F1 | GCA_026122975 |
| *Ca*. Electronema palustre F3 | GCA_026122955 |
| SI2 | PQ214939 |
| *Ca*. Electronema sp. YR | PQ214942 |
| *Ca*. Electronema sp. SI1 | PQ214940 |
| GU208270 | GU208270 |
| JF268391 | JF268391 |
| KR814208 | KR814208 |
| *Ca*. Electronema sp. NC1 | PQ351298 |
| *Ca*. Electronema sp. NC2 | PQ351299 |
| *Desulfobulbus propionicus* DSM2032 | CP002364 |


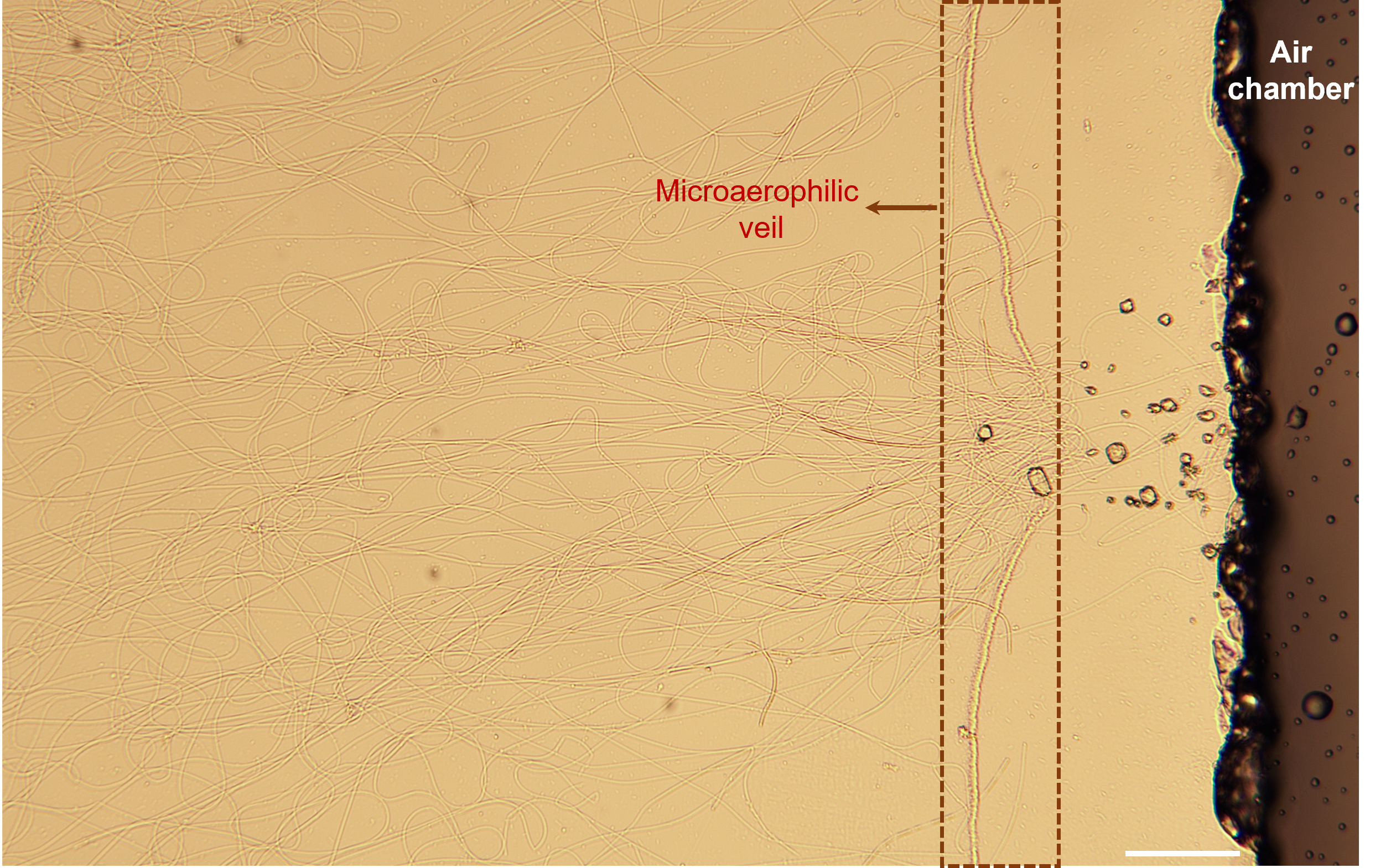


**Fig S1.** Observation of a microaerophilic veil on the microchamber slide. Within 48 hours, cable bacteria filaments emerge from the sediment chamber and move towards the air chamber. Aerobic microorganisms formed a microaerophilic veil at a distance of 200-300 μm from the air chamber. Scale bar: 100 µm.


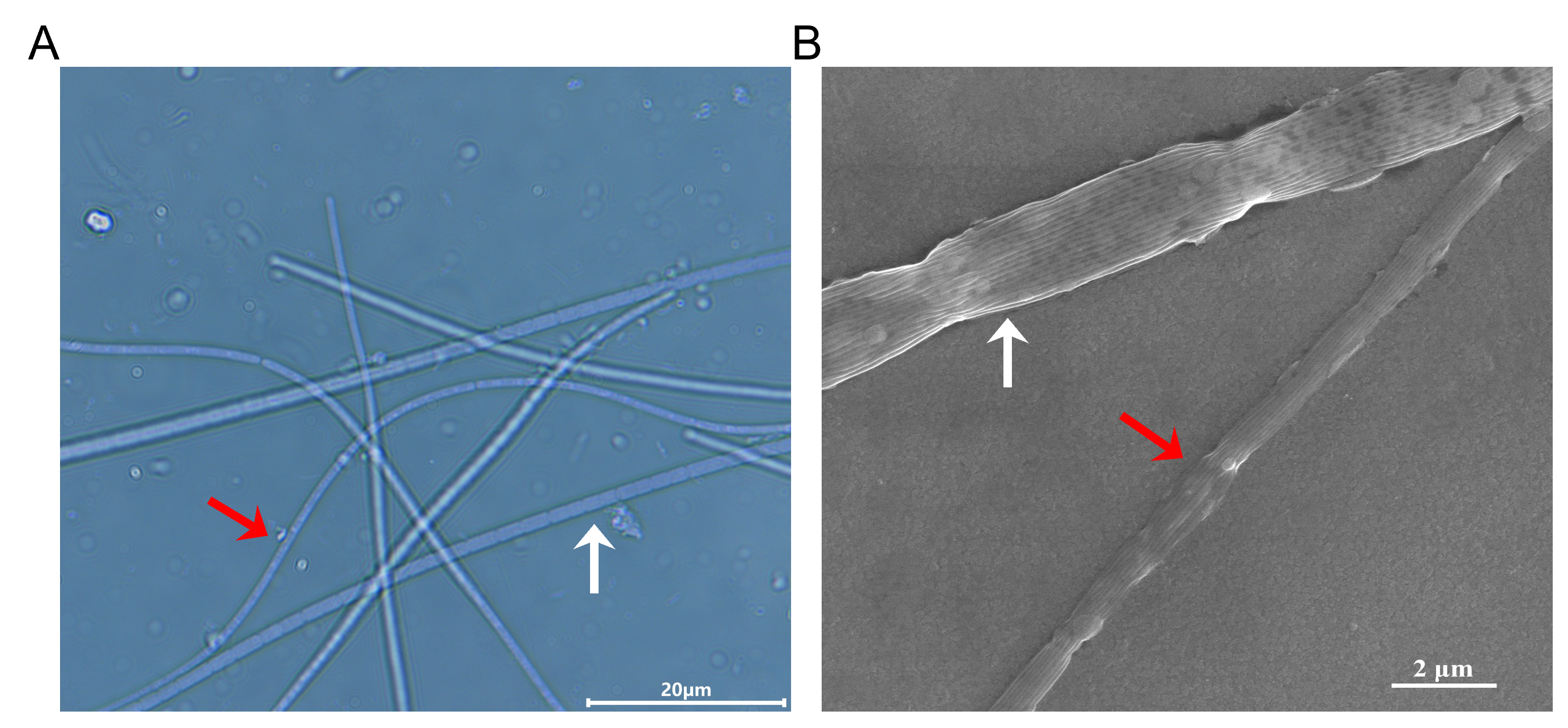


**Fig S2.** Bright field (A) and SEM (B) microscopy show that cable bacterium filaments in the slide setup exhibit two different morphological types with different diameters. The red arrow indicates the thinner cable bacteria filaments, whereas the white arrow marks the thicker one filaments.


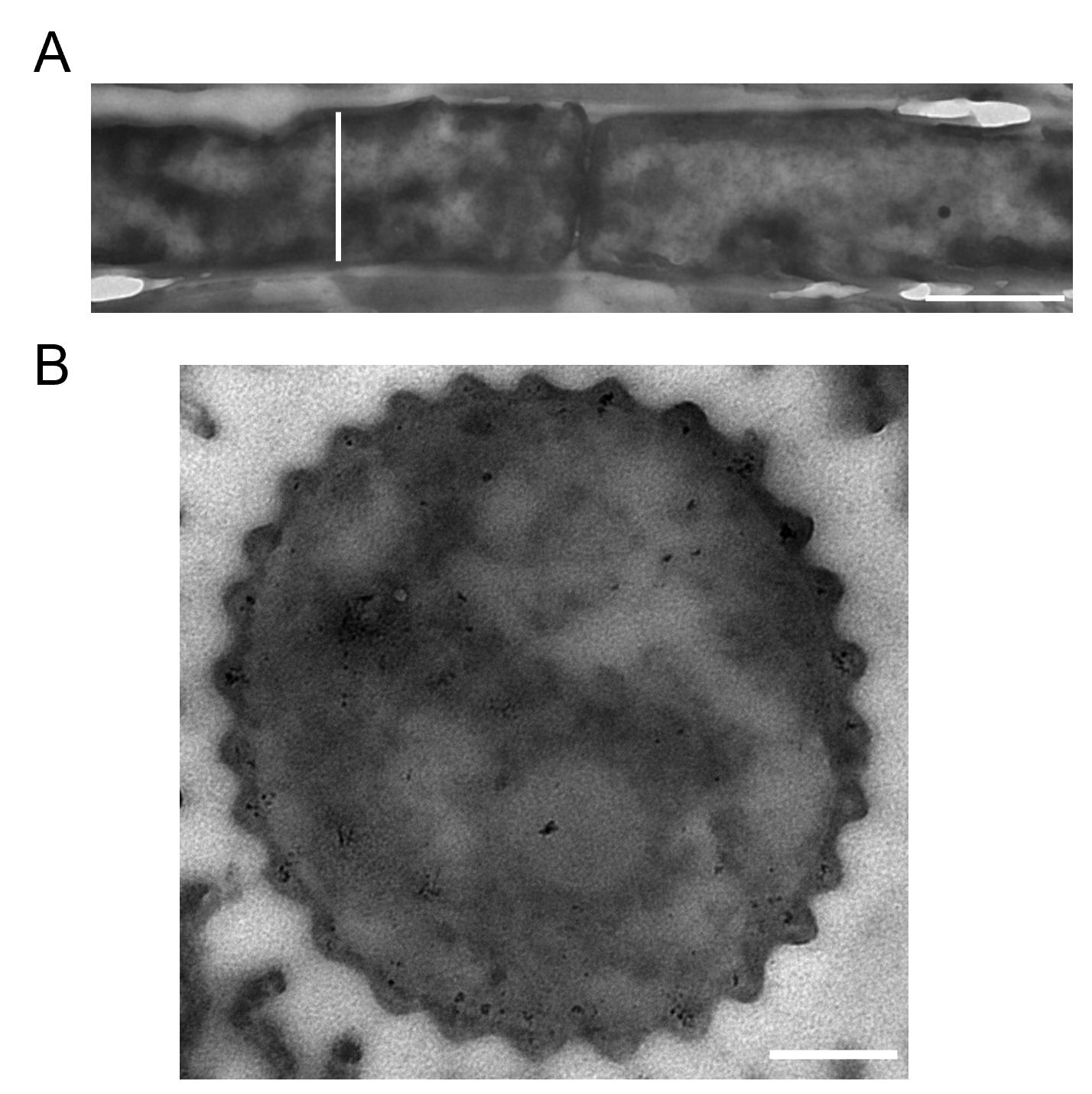


**Fig S3.** Transmission electron microscope (TEM) images of thin cross-sections of resin-embedded cable bacteria filaments. (A) Longitudinal cross section. The white line indicates the location of the transverse cross-section in B. Scale bar: 1 µm; (B) Transversal cross section. The filament has an outer diameter of 1 µm and shows 28 semicircular ridges. Scale bar: 200 nm.


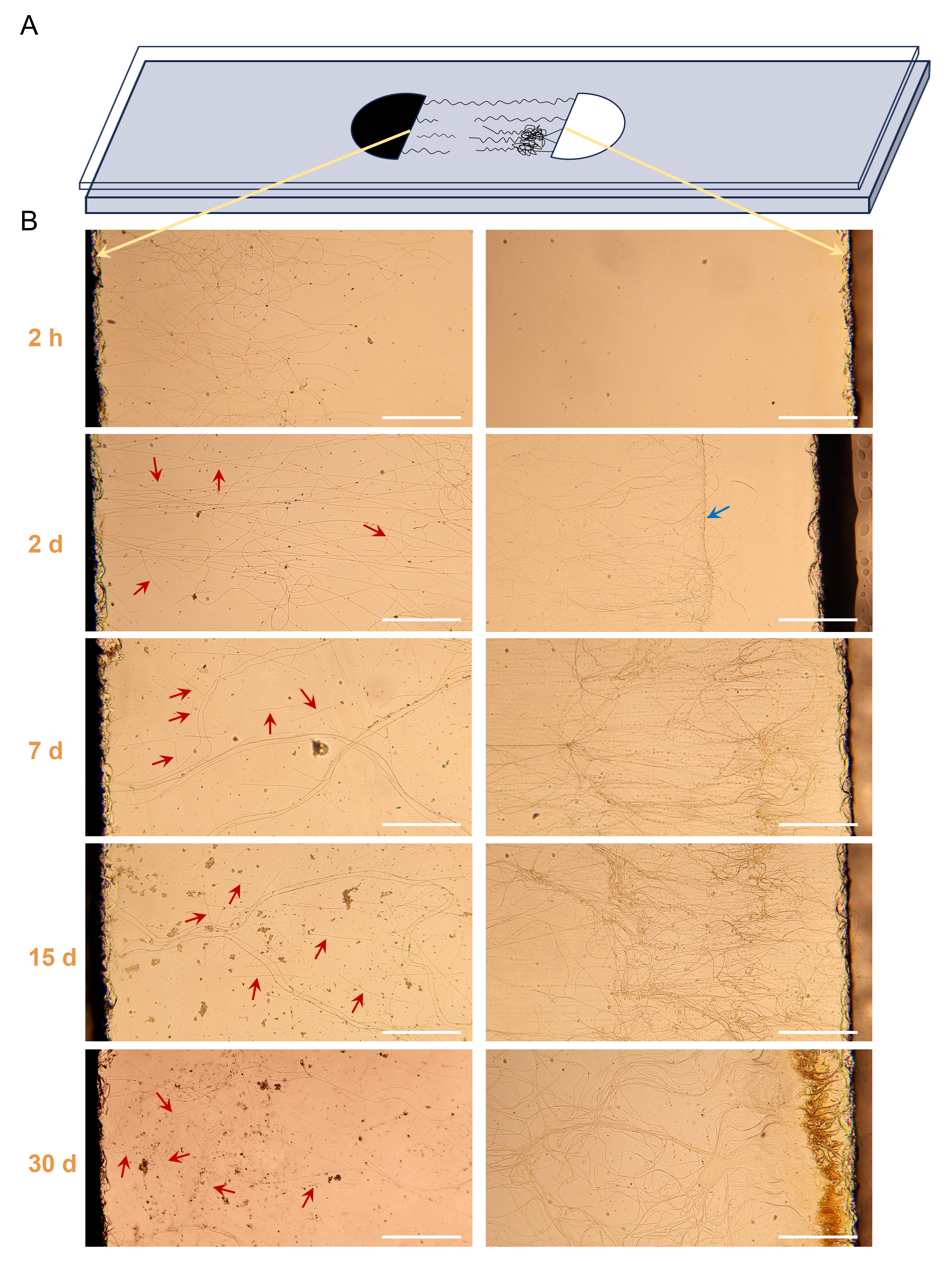


**Fig S4.** The migration and positioning cable bacterium filaments during 30 days of incubation in the slide setup. (A) Schematic diagram of the filament distribution in the slide (after 7 days). (B) The distribution of filaments near the sediment chamber (left images) and the air chamber (right images) at different incubation times (2 h, 2 d, 7 d, 15 d, 30 d). Short filaments near the sediment chamber are marked with red arrows, and the microaerophilic veil near the air chamber is indicated by a blue arrow. Scale bars: 200 µm.


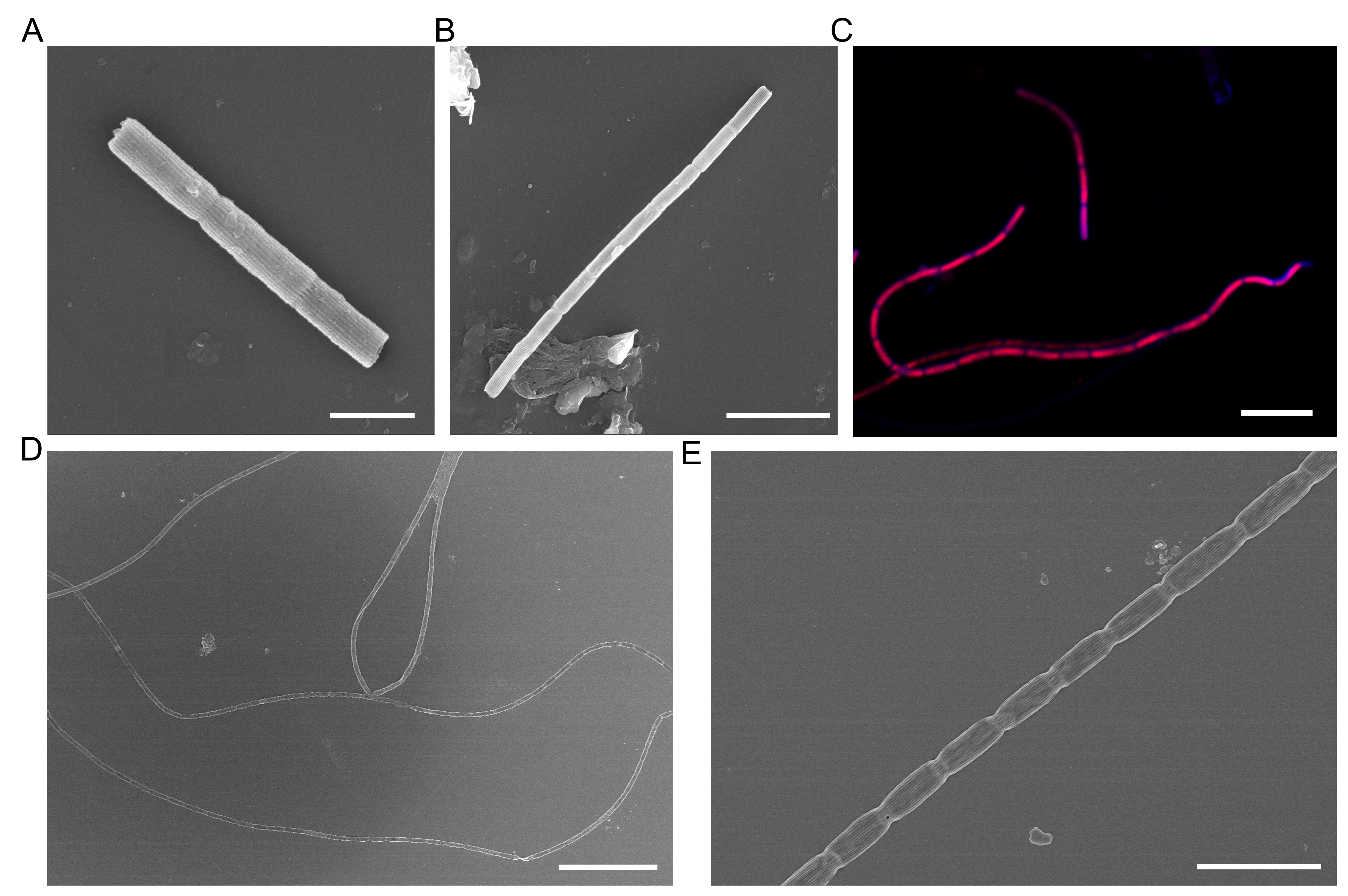


**Fig S5.** Characterization of filaments near the sediment and air chambers. (A) SEM image of short filament near the sediment chamber. Scale bar: 2 µm. (B) SEM image of another short filament near the sediment chamber. Scale bar: 5 µm. (C) FISH identification reveals that short filaments near the sediment chamber are cable bacteria. Scale bar: 10 µm. (D) SEM image of long cable bacteria near the air chamber. Scale bar: 20 µm. (E) SEM image of another long filament near the air chamber. Scale bar: 5 µm.


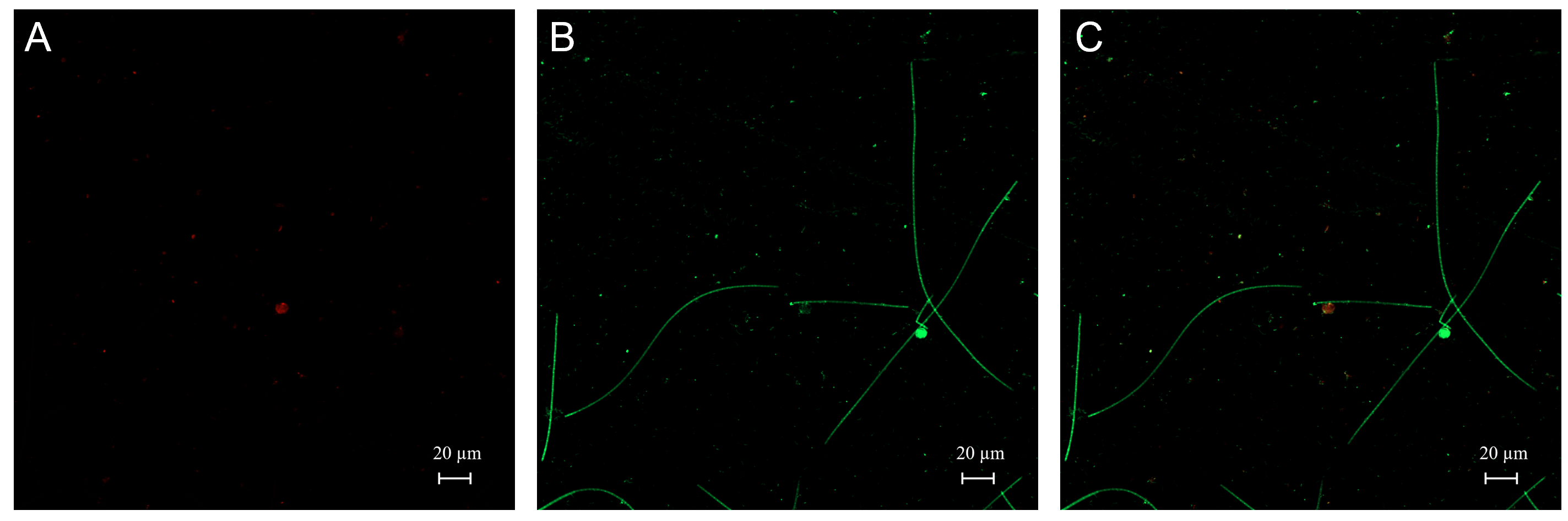


**Fig S6.** Confocal scanning light microscope images of Live/dead stained short cable bacterium filaments. (A) Red channel, (B) green channel, and (C) overlay of panels A and B. The red fluorescence identifies dead cells, whereas the green fluorescence signifies viable cells.


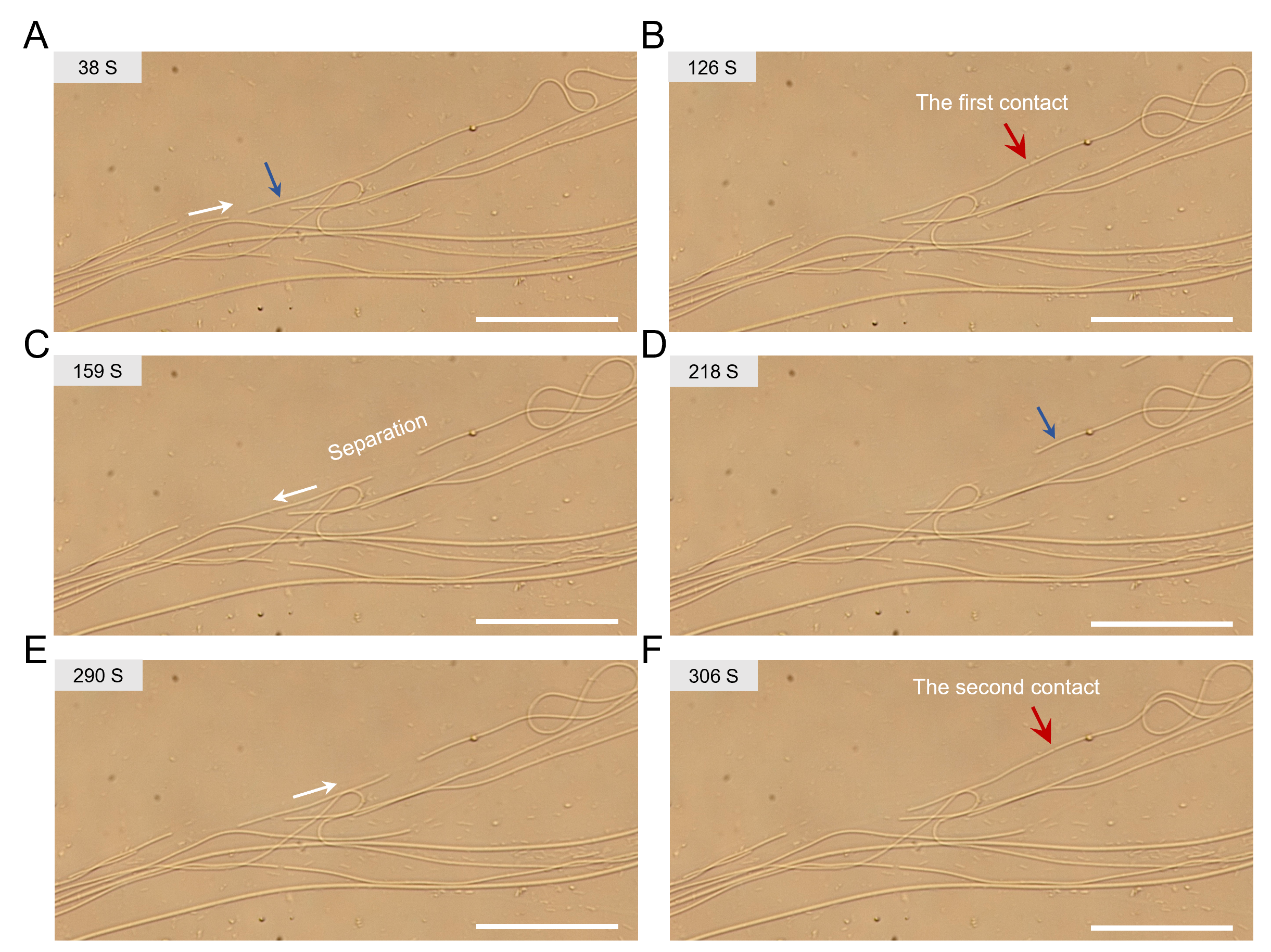


**Fig S7.** The repeated contact and separation behavior of two cable bacteria filaments (6 stills taken from Movie S4). (A) A short cable bacteria filament (white arrow) moves from the sediment towards a long cable bacteria filament (blue arrow) that is connected on the air chamber. (B) The first contact is completed (red arrow). (C) The separation begins, and the short filament moves toward the sediment chamber. (D) The short filament has migrated out of view, while the long filament (blue arrow) remains in place. (E) The short filament moves back towards the long filaments and follows the same trajectory. (F) The second contact is made. Scale bars: 50 μm.


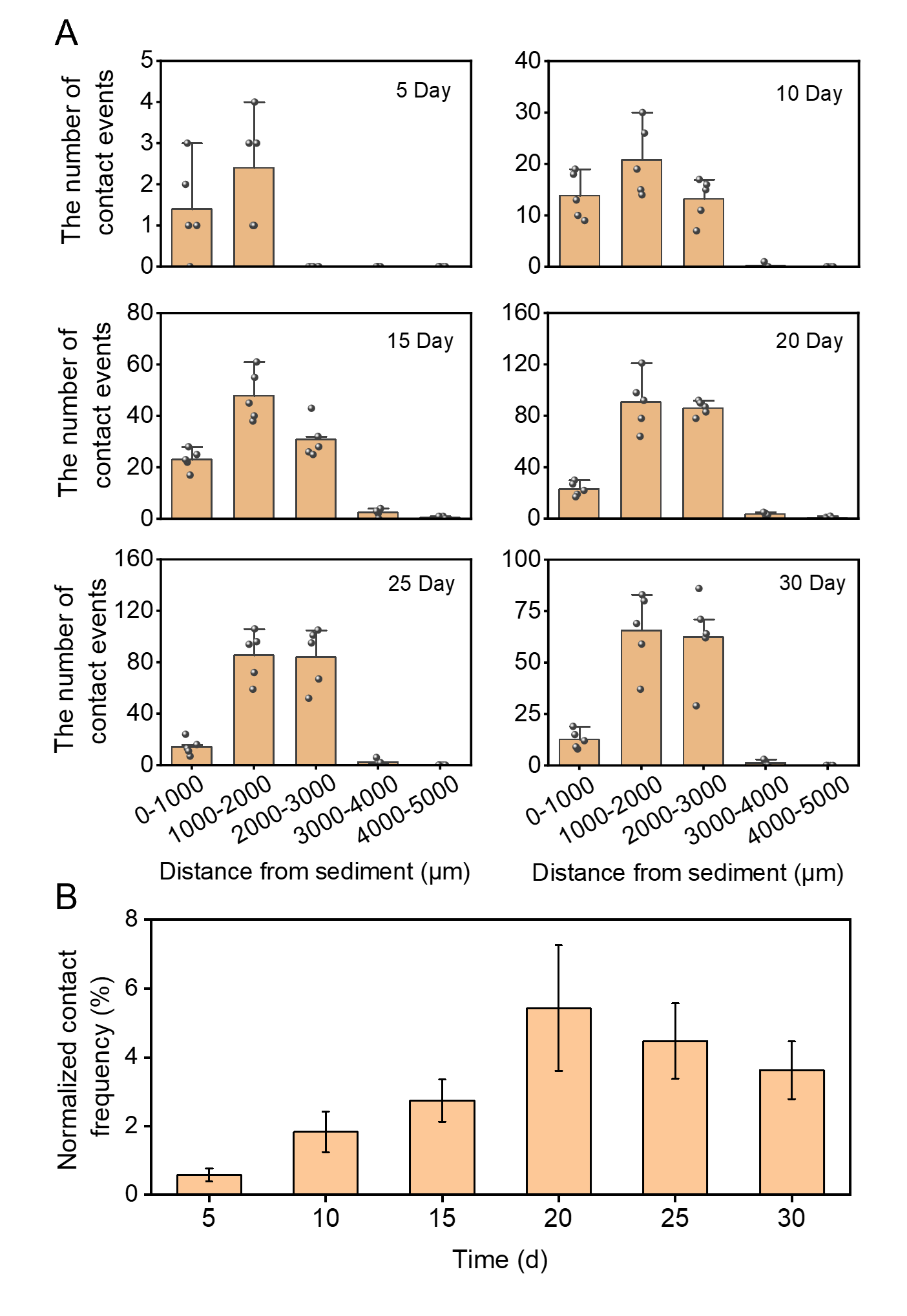


**Fig S8.** Contact frequency and spatial distribution of contact sites. (A) Distribution of inter-filament contact sites in the 5 mm gap between the sediment chamber and the air chamber at different incubation times (5, 10, 15, 20, 25, 30 days). (B) Temporal variation of contact frequency over the incubation experiment (mean and standard deviation over n = 5 microchamber set-ups). Contact frequency is calculated by dividing the number of contact events over a 2-hour observation period by the total number of filaments observed in microscope slide setup.

**
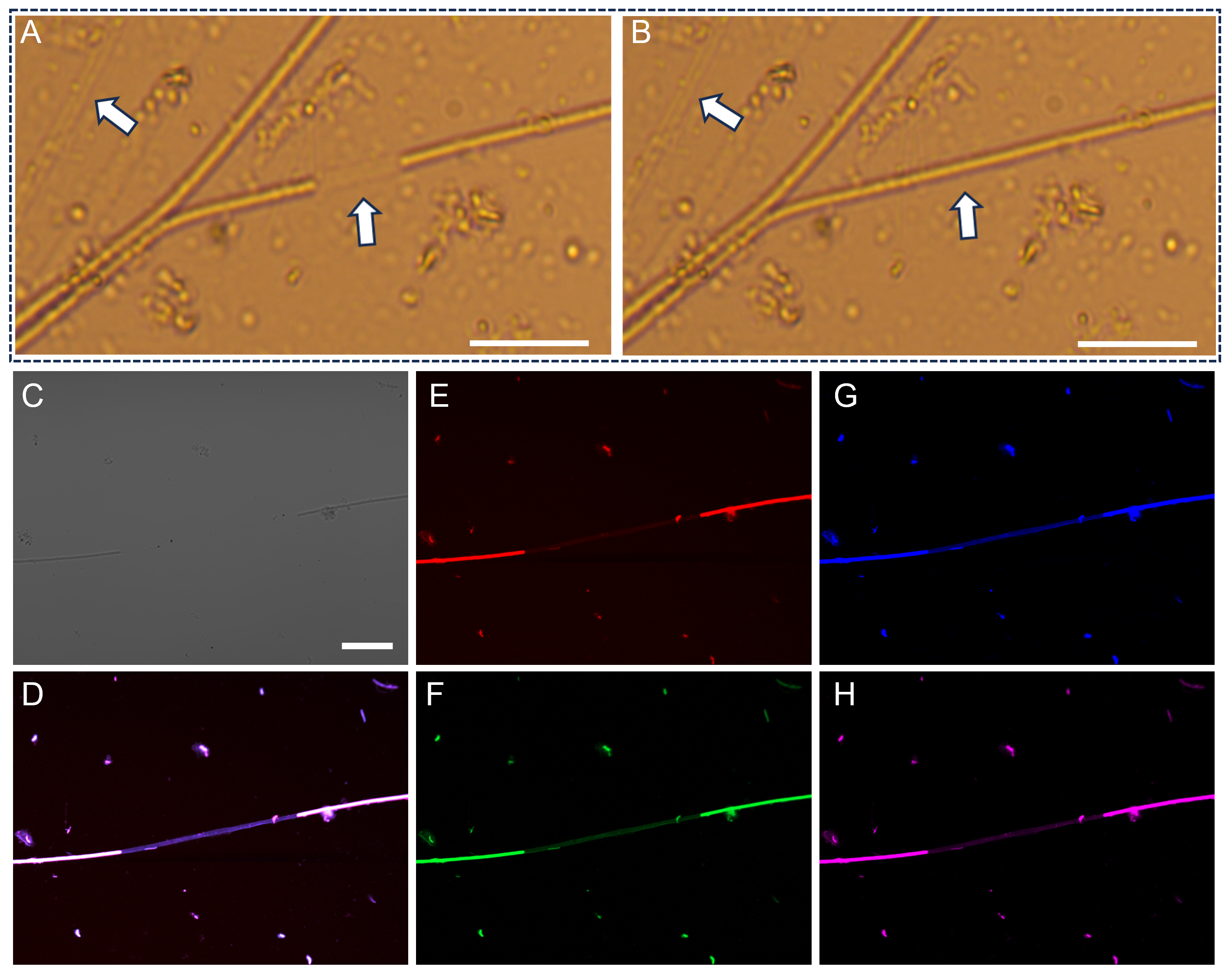
**

**Fig S9.** Visualization of extracellular polymeric substances (EPS) during cable bacteria interaction. (A-B) Bright field images of cable bacterium filaments before (A) and after (B) inter-filament contact. Arrows indicate the presence of EPS trails. (C) CLSM bright-field image showing two cable bacterial filaments connected by an EPS trail (the latter is hardly visible). Scale bar: 20 µm. (D) Composite image of different images shown in (E)–(H). (E) CLSM fluorescence image after Nile red staining targeting lipids. (F) CLSM fluorescence image of FITC staining targeting proteins. (G) CLSM fluorescence image after Calcofluor white staining targeting β-D-glucopyranose polysaccharides. (H) CLSM fluorescence image after Con A staining targeting α-D-glucopyranose polysaccharides.

**Movie S1.** The movement behaviors of cable bacteria. The time-lapse movie shows cable bacteria emerging from the sediment (left) and migrating toward oxygen (right) in the microchamber slide. The 40-minute process is compressed to 17 seconds for visualization. Scale bar: 200 μm.

**Movie S2.** The behaviors of contact and separation of cable bacteria under microscope dark-field mode. White/yellow/red arrows denote sulfide/oxygen directions, bacterial movement, and contact sites. Time compressed from 40 min to 43 s. Scale bar: 50 μm.

**Movie S3.** The nonspecific contact behaviors of cable bacteria. The time-lapse movie shows the cable bacteria near the oxygen side contacting with two different cable bacteria near the sulfide side. The white arrows indicate the directions of sulfide (left) and oxygen (right), respectively. The yellow arrows indicate the direction of cable bacteria movement, and the red arrows indicate the contact sites. Time compressed from 60 min to 46 s (20-40 s at 3 × speed). Scale bar: 50 μm.

**Movie S4.** The cyclical contact and separation behaviors of two individual filaments. The time-lapse video shows that one filament move toward the sulfide side after completing contact until it disappear from view, and then return to the oxygen side to re-contact. The white arrows indicate the directions of sulfide (left) and oxygen (right), respectively. The yellow arrows indicate the direction of cable bacteria movement, and the red arrows indicate the contact sites. Time is compressed from 312 s to 57 s (35-45 s at 3 × speed). Scale bar: 50 μm.

**Reference**

[1] van der Veen JR, Valianti S, van der Zant HS et al. A model analysis of centimeter-long electron transport in cable bacteria. *Phys. Chem. Chem. Phys* 2024;**26**:3139-3151.

[2] Geelhoed J S, Van de Velde S J, Meysman F J R. Quantification of cable bacteria in marine sediments via qPCR. *Front. Microbiol* 2020;**11**:1506.

[3] Meysman FJR, Cornelissen R, Trashin S et al. A highly conductive fibre network enables centimetre-scale electron transport in multicellular cable bacteria. *Nat. Commun* 2019;**10**:4120.
